# Comparing methods for identifying patients with heart failure using electronic data sources

**DOI:** 10.1186/1472-6963-9-237

**Published:** 2009-12-18

**Authors:** Fadi Alqaisi, L Keoki Williams, Edward L Peterson, David E Lanfear

**Affiliations:** 1 Henry Ford Heart and Vascular Institute, Henry Ford Hospital, 2799 W. Grand Blvd., Detroit, MI 48202 USA; 2 Center for Health Services Research, Henry Ford Hospital, One Ford Place, Detroit, MI 48202 USA; 3 Biostatistics and Research Epidemiology, Henry Ford Hospital, One Ford Place, Detroit, MI 48202 USA

## Abstract

**Background:**

Accurately indentifying heart failure (HF) patients from administrative claims data is useful for both research and quality of care efforts. Yet, there are few comparisons of the various claims data criteria (also known as claims signatures) for identifying HF patients. We compared various HF claim signatures to assess their relative accuracy.

**Methods:**

In this retrospective study, we identified 4174 patients who received care from a large health system in southeast Michigan and who had ≥1 HF encounter between January 1, 2004 and December 31, 2005. Four hundred patients were chosen at random and a detailed chart review was performed to assess which met the Framingham HF criteria. The sample was divided into 300 subjects for derivation and 100 subjects for validation. Sensitivity, specificity,, and area under the curve (AUC) were determined for the various claim signatures. The criteria with the highest AUC were retested in the validation set.

**Results:**

Of the 400 patients sampled, 65% met Framingham HF criteria, and 56% had at least one B-type Natriuretic Peptide (BNP) measurement. There was substantial variation between claims signatures in terms of sensitivity (range 15%-77%) and specificity (range 69%-100%). The best performing criteria in the derivation set was if patients met any one of the following: ≥2 HF encounters, any hospital discharge diagnosis of HF, or a BNP ≥200 pg/ml. These criteria showed a sensitivity of 76%, specificity of 75%, and AUC of 0.754 for meeting the Framingham HF criteria. This claims signature performed similarly in the validation set.

**Conclusion:**

Claim signatures for HF vary greatly in their relative sensitivity and specificity. These findings may facilitate efforts to identify HF patients for research and quality improvement efforts.

## Background

Heart failure (HF) is a major public health problem in the United States. Approximately 5 million patients have HF, and over 550,000 patients are diagnosed with HF for the first time each year [1]. It is the fifth ranking cause for hospitalization overall, and a leading cause for hospitalization in the elderly [1].

Many quality initiatives and clinical research efforts rely on administrative claims data to identify patients with HF. Most often this involves identifying individuals coded with a diagnosis of HF according to International Classification of Diseases 9 (ICD9) codes. While claims data are widely available, their utility is limited by the fact that encounters coded as HF may not reflect accepted epidemiologic criteria. To try to improve the utility of administrative data, various claim signatures using combinations of specific codes, such as multiple encounters or particular encounter types (e.g., hospitalizations) have been utilized.

Unfortunately, even by restricting code types (i.e. inpatient vs. outpatient, primary vs. secondary) or using combinations of codes identification of HF patients using administrative data is imperfect. For example, one study at our institution showed that more than a third of patients identified as having HF by administrative codes did not satisfy Framingham HF criteria [2]. On the other hand, claim signatures with greater specificity, such as those requiring a primary hospital discharge diagnosis of HF, have been shown to result in samples in which >90% of subjects meet the Framingham HF definition [3]. However, this latter approach is likely at great cost to sensitivity, thereby under-identifying patients with known HF. Perhaps more problematic is the paucity of data comparing such criteria in order to provide some estimate of their performance. In addition to claims data, many health systems have access to electronic laboratory results which can be queried. Included in these laboratory data may be BNP measurement. These levels have been shown to be helpful in differentiating HF from other causes of dyspnea [4]. Therefore, augmenting claims data with laboratory data, specifically BNP levels, may be useful in identifying HF patients from electronic data sources.

In this study, we sought to compare the relative accuracy of various claim signatures for HF using both administrative claims data and laboratory BNP levels by testing them against a widely used HF criterion.

## Methods

### Study Population and Setting

This study was approved by the Institutional Review Board at Henry Ford Health System. The study was also in compliance with the health system's Health Insurance Portability and Accountability Act policy. Patients were both members of a large, health maintenance organization (HMO) in southeast Michigan and received their care from a large, multi-specialty medical group. To be included in the cohort, patients had to be age ≥18 years and have at least one encounter code for HF (ICD9 codes 428.xx, 398.91, 402.01, 402.11, or 402.91) between January 1, 2004 and December 31, 2005 (excluding all emergency department encounters). In order to minimize misclassification of patients as having heart failure we excluded patients with a diagnosis of end-stage renal disease or chronic obstructive pulmonary disease with steroid use as these conditions can manifest symptoms confused with heart failure. We identified end stage renal disease by encounter ICD-9 codes (585, 586, V42.0, 996.81) or procedure codes (M0916, M0920, M0932, M0936, M0937, M0945, M0974, M0978, M0982, W6800, X3732, 36800, 3681A, 3681B, 3681C, 36810, 36815, 3683C, 3683E, 36833, 90925, 90935, 90937, 90941, 90942, 90943, 90944, 90945, 90947, 90951, 90952, 90953, 90954, 90955, 90956, 90957, 90958, 90966, 90967, 90969, 90976, 90977, 90978, 90979, 90982, 90983, 90984, 90985, 90989, 9099C, 9099D, 9099E, 9099F, 90993, 90999, 90921, 90919, A4726, A4656, 90922, A4722, 50360, 50365, 50370, 50380). Chronic obstructive pulmonary disease was identified by encounter ICD-9 code (490, 491, 492, 493, 494, 495, 496, 500, 501, 502, 503, 504, or 505) and oral steroid use (i.e., a pharmacy claim for a fill of prednisone or NDA category P5A) over the time period from January 1, 2004 through December 31, 2005. We identified 4,174 patients from which we randomly selected a sample of 400 patients to have a detailed medical chart review.

### Endpoint Assessment

A physician reviewed each medical chart in detail and documented the presence or absence of major and minor Framingham HF criteria [5]. Patients were classified as having satisfied the Framingham definition for HF if they met either two major criteria or one major and two minor criteria. Patients were also categorized according to 18 different case-definitions using claims and laboratory data. These latter definitions used various combinations of HF coded encounters (e.g., ambulatory setting, hospital setting, primary diagnosis, secondary diagnoses, but excluded any emergency department encounters) and BNP levels. For the purpose of this study we define a *claims signature *as any definition using a combination of these data queried electronically.

### Statistical Analysis

The cohort of 400 patients was divided into 300 subjects for derivation testing, and 100 subjects were used to validate the top performing claims signature definitions. The Framingham HF definition was chosen as the comparator criteria as it has been widely used in previous studies. Sensitivity, specificity, likelihood ratios, and area under the curve (AUC) were determined for each claim signature as compared to classification using Framingham criteria. The claims signatures with the highest AUC were retested in the validation set. All calculations were performed using SAS 9.1.3 (SAS Institute Inc.)

## Results

A total of 4,174 patients had at least one encounter code for HF. From this population, a sample of 400 patients was randomly chosen for further examination. This group of 400 patients was further divided into a derivation set (n = 300) and a validation set (n = 100). The characteristics of subjects in the derivation set, validation set, and remaining patient parent population are shown in Table 1. These characteristics were similar across groups except for the prevalence of coronary disease which was higher in unselected patients compared to the randomly selected study cohort (20% vs. 14% vs. 15%, p = 0.012). Overall, 65% of subjects in the study sample met the Framingham definition of HF. Fifty seven percent of the total cohort and 56% of the study sample had at least one BNP measurement.

**Table 1:** Characteristics of patients with at least one encounter for heart failure between January 1, 2004 and December 31, 2005 stratified by their participation in the study derivation and validation sets.

Variable	Total Population (n = 4,174)	Derivation sample (n = 300)	Validation sample (n = 100)	Unselected patients (n = 3,774)	P-value*
Age (years) -- mean ± SD	67 ± 14	67 ± 14	68 ± 14	67 ± 14	0.47

Male -- no.(%)	2011 (48%)	155 (52%)	46 (46%)	1810 (48%)	0.42

White -- no. (%)	2615 (63%)	61%	59%	63%	0.12

Hypertension -- no. (%)	3384 (81%)	244 (81%)	77 (77%)	3063 (81%)	0.57

Diabetes -- no. (%)	1409 (34%)	104 (35%)	31 (31%)	1274 (34%)	0.80

Chronic obstructive pulmonary disease - no. (%)	600 (14%)	46 (15%)	12 (12%)	542 (14%)	0.71

Coronary Atherosclerosis - no. (%)	829 (20%)	42 (14%)	15 (15%)	772 (20%)	0.012

≥1 BNP measurement during the study period - no. (%)	2374 (57%)	170 (57%)	53 (53%)	2151 (57%)	0.73

Maximum BNP level - median (interquartile range)	198 (88-494)	192 (84-500)	144 (90-379)	201 (89-500)	0.634

SD denotes standard deviation

*P-value for the comparison across patients in the derivation set, patients in the validation set, and patients not used in the analysis.

Calculated sensitivity, specificity, and area under curve (AUC) for each claims signature relative to the Framingham HF definition is shown in Table 2. The best performing case-definition was the following combination: ≥2 outpatient encounters for HF, any hospital discharge diagnosis of HF, or any single BNP ≥200 pg/ml. These criteria had a sensitivity of 76.3%, specificity of 74.5%, positive likelihood ratio of 3.0, negative likelihood ratio of 0.32, and an AUC of 0.754 for meeting the Framingham HF criteria. When retested in the validation patient sample, this definition performed similarly with sensitivity of 78%, a specificity of 69%, and an AUC of 0.735. Since this validation AUC was lower than three other definitions from the derivation set, we also retested these three definitions in the validation set (Table 3). The obtained AUCs were 0.725, 0.730 and 0.711 respectively, consistent with the relative performance of the derivation set.

**Table 2:** Performance of various definitions for heart failure in derivation set (n = 300)*

#	Heart failure criteria based on claims and laboratory data	Sensitivity	Specificity	AUC
1	≥2 heart failure encounters OR any hospital discharge diagnosis of heart failure OR BNP ≥ 200 pg/ml	76.29	74.53	0.754

2	≥2 heart failure encounters OR any hospital discharge diagnosis of heart failure	68.56	81.13	0.748

3	≥2 heart failure encounters OR BNP ≥ 200 pg/ml	69.59	79.25	0.744

4	≥2 heart failure encounters OR ***any hospital discharge diagnosis of heart failure OR ***BNP ≥ 500 pg/ml	69.59	78.3	0.739

5	≥2 heart failure encounters OR primary hospital discharge diagnosis of heart failure	60.31	85.85	0.729

6	≥2 heart failure encounters OR BNP ≥ 100 pg/ml	76.29	68.87	0.726

7	≥2 heart failure encounters	58.76	85.85	0.723

8	≥2 heart failure encounters AND ≥ 1 BNP ≥ 50 pg/ml OR any hospital discharge diagnosis of heart failure OR ≥ 1 BNP ≥ 200 pg/ml	59.28	81.13	0.702

9	≥2 heart failure encounters AND ≥ 1 BNP ≥ 50 pg/ml OR primary hospital discharge diagnosis of heart failure OR ≥ 1 BNP ≥ 500 pg/ml	38.66	90.57	0.646

10	Any hospital discharge diagnosis of heart failure OR BNP ≥ 500 pg/ml	40.72	87.74	0.642

11	Any hospital discharge diagnosis of heart failure	35.05	91.51	0.633

12	BNP ≥ 100 pg/ml	48.97	76.42	0.627

13	BNP ≥ 200 pg/ml	36.08	87.74	0.619

14	Primary hospital discharge diagnosis of heart failure OR BNP ≥ 500 pg/ml	24.23	95.28	0.598

15	BNP ≥ 500 pg/ml	20.1	95.28	0.577

16	Any primary hospital discharge diagnosis of heart failure	14.95	100	0.575

*Calculated sensitivity, specificity, and area under curve for each claim signature is compared to the Framingham definition of heart failure (i.e., meeting either two major criteria or one major and two minor criteria).

**Table 3:** Replication of definitions #1 - #4 in validation set of patients (n = 100)*

#	Heart failure criteria based on claims and laboratory data	Sensitivity (%)	Specificity (%)	*AUC*
1	≥2 heart failure encounters OR any hospital discharge diagnosis of heart failure OR ≥ 1 BNP level ≥ 200 pg/ml	78.46	68.57	0.735

2	≥2 heart failure encounters OR any hospital discharge diagnosis of heart failure	70.77	74.29	0.725

3	≥2 heart failure encounters OR ≥ 1 BNP level ≥ 200 pg/ml	63.08	82.86	0.730

4	≥2 heart failure encounters OR ***any hospital discharge diagnosis of heart failure OR ***≥ 1 BNP ≥ 500 pg/ml	70.77	71.43	0.711

*Calculated sensitivity, specificity, and area under curve for each claim signature is compared to the Framingham definition of heart failure (i.e., meeting either two major criteria or one major and two minor criteria).

As electronically searchable laboratory data may not be available in other health systems, we examined the best performing definition using only claims data. The best performing of these criteria was as follows: ≥2 HF ambulatory encounters or any hospital discharge (definition #2 in Table 2). This combined definition had a sensitivity of 69%, a specificity of 81%, and an AUC of 0.748 when compared with the Framingham HF criteria, and performed similarly in validation (see Table 3). When comparing this definition to the best one employing BNP measures, there was only a modest increase in the AUC between the two criteria (0.748 vs. 0.754, respectively); however, this difference was statistically significant (P value = 0.02).

## Discussion

Administrative data have inherent limitations in terms of accuracy but may be a valuable resource because of their wide availability and the potentially large number of subjects that can be identified through their use. To our knowledge, this is the first study to formally compare different criteria for HF using electronic data sources. In so doing, we found that our best performing criteria had an AUC of 0.754, and maintained a reasonable sensitivity and specificity of 76.3% and 74.5%. We realize that the tradeoff between sensitivity and specificity depends on the purpose for which these data are to be used. For example, projects focusing on the quality of HF care may want to be confident subjects actually have HF and thus may select the highest specificity criteria, while another project with the objective of identifying the probable size of the affected population may opt for a criterion with high sensitivity. Therefore, we anticipate that the range of claims signatures that we present may be helpful to broad variety of users, such as researchers, chronic care managers, and providers.

While there appears to be a paucity of studies assessing the accuracy of claims signatures of heart failure, our data fit well into the context of existing data. As pointed out above, a hospital discharge with primary diagnosis of HF has been shown to be quite specific for heart failure [3]. Our data supported this, showing 100% specificity, but also added further insight by revealing that the associated sensitivity was extremely low at only 15%. A previous study from our institution using the claim signature of two outpatient encounters or any hospital discharge for HF found that the resulting cohort failed to meet Framingham HF criteria [2]. In contrast, in our data set using the same criteria, 87% of subjects met Framingham definition for HF. This could suggest that physician diagnosis and coding for heart failure has improved over time since the prior analysis was from 1989-1999 and ours from 2004-2005. Alternatively this could be due to the mistaken inclusion of more patients with renal disease in the former study, since its criteria included cardiovascular codes with chronic kidney disease. This suggests that some patients may have been coded (using a single ICD9 code) as having both renal failure *and *HF but upon closer scrutiny may not have met objective criteria for HF. There is one previous study assessing the under-diagnosis and/or under-coding of HF among patients with coronary disease [6]. While HF is likely under recognized in the community, our study did not address this important issue. Rather it was focused on another essential task, identifying likely HF patients from among those so coded, since many do not meet standard criteria.

These data have several additional limitations that should be noted. First, our study was performed at a single health system, and therefore our results may differ from other systems. However, our covered patient population is generally reflective of the population of southeast Michigan, and it is demographically diverse in terms of race-ethnicity and socioeconomic status. Second, we initially selected patients who had at least one HF encounter. While this assuredly increased the prevalence of true HF cases in the sample (i.e., 65% of the study sample met Framingham criteria for HF), we felt this was the most practical approach since sampling from the total population (~150,000 covered lives) would have likely resulted in a very small proportion of HF cases and therefore would have required samples sizes beyond the scope of the current project to discern differences between claim signatures. Because of our approach, we could not assess for physician under-diagnosis of HF, as has been addressed elsewhere [6]. Still, this should be considered as a limitation of our approach, specifically that patients who did not meet Framingham criteria may still have actual HF and by selecting subjects who had at least one code for HF this could have been amplified. As such, our claims signatures are unlikely to be able to rule out the existence of HF. Yet HF patients without at least one diagnosis code for HF are unlikely to be reliably identified through other administrative data (i.e., other diagnostic codes or laboratory values) and therefore would be an impractical population for whom to develop a claims signature. Another limitation is that there was a difference in the prevalence of coronary disease between the study cohort and the broader source population. This selection bias could in theory effect the results, however the direction and magnitude of this bias is unknown. Finally, there is no universally recognized 'best' definition for HF [7], and several other validated criteria exist such as the Boston [8], NHANES [9] and Minnesota criteria [7]. While some of these may perform better than the Framingham criteria, the latter continue to be widely recognized and utilized [7,10,11]. Utilizing a different set of HF criteria could have resulted in different absolute estimates of sensitivity and specificity.

## Conclusions

In summary, we observed considerable variation in the sensitivity and specificity of the various criteria for heart failure using electronic data sources. However, we did identify a best performing combination of diagnoses and laboratory results which showed consistent performance in validation. We hope that these findings may facilitate efforts to identify HF patients for research and quality improvement efforts, or at minimum encourage others to consider this variation when deciding on criteria to identify HF patients in their own patient population.

## Competing interests

The authors declare that they have no competing interests.

## Authors' contributions

All authors have read and approve of the final manuscript. FA was responsible for data collection, interpretation, and drafting of the manuscript. EP was responsible for data analysis and critical revision of the manuscript. KW was responsible for study design, interpretation, and critical revision of the manuscript. DL was responsible for study design, data analysis, interpretation, and drafting the manuscript.

## Pre-publication history

The pre-publication history for this paper can be accessed here:

http://www.biomedcentral.com/1472-6963/9/237/prepub
